# Hybrid elementary flux analysis/nonparametric modeling: application for bioprocess control

**DOI:** 10.1186/1471-2105-8-30

**Published:** 2007-01-29

**Authors:** Ana P Teixeira, Carlos Alves, Paula M Alves, Manuel JT Carrondo, Rui Oliveira

**Affiliations:** 1 IBET/ITQB, Apartado 12, P-2781-901 Oeiras, Portugal; 2 REQUIMTE, Laboratório de Engenharia Bioquímica, FCT/UNL P-2829-516 Caparica, Portugal; 3 Laboratório de Engenharia Bioquímica, FCT/UNL P-2829-516 Caparica, Portugal

## Abstract

**Background:**

The progress in the "-omic" sciences has allowed a deeper knowledge on many biological systems with industrial interest. This knowledge is still rarely used for advanced bioprocess monitoring and control at the bioreactor level. In this work, a bioprocess control method is presented, which is designed on the basis of the metabolic network of the organism under consideration. The bioprocess dynamics are formulated using hybrid rigorous/data driven systems and its inherent structure is defined by the metabolism elementary modes.

**Results:**

The metabolic network of the system under study is decomposed into elementary modes (EMs), which are the simplest paths able to operate coherently in steady-state. A reduced reaction mechanism in the form of simplified reactions connecting substrates with end-products is obtained. A dynamical hybrid system integrating material balance equations, EMs reactions stoichiometry and kinetics was formulated. EMs kinetics were defined as the product of two terms: a mechanistic/empirical known term and an unknown term that must be identified from data, in a process optimisation perspective. This approach allows the quantification of fluxes carried by individual elementary modes which is of great help to identify dominant pathways as a function of environmental conditions. The methodology was employed to analyse experimental data of recombinant Baby Hamster Kidney (BHK-21A) cultures producing a recombinant fusion glycoprotein. The identified EMs kinetics demonstrated typical glucose and glutamine metabolic responses during cell growth and IgG1-IL2 synthesis. Finally, an online optimisation study was conducted in which the optimal feeding strategies of glucose and glutamine were calculated after re-estimation of model parameters at each sampling time. An improvement in the final product concentration was obtained as a result of this online optimisation.

**Conclusion:**

The main contribution of this work is a novel bioreactor optimal control method that uses detailed information concerning the metabolism of the underlying biological system. Moreover, the method allows the identification of structural modifications in metabolism over batch time.

## Background

Knowledge of intracellular metabolic fluxes is crucial to understand how different pathways interact and their relative importance within the overall metabolic processes. Metabolic flux analysis (MFA) is an established methodology that allows the quantification of such intracellular fluxes. In MFA, intracellular fluxes are calculated by applying steady-state material balances around intracellular metabolites. In general the number of reactions exceeds the number of metabolites resulting in undetermined systems of algebraic equations [1]. Such systems can be solved after measurement of the missing fluxes, which are typically uptake rates of substrates and secretion rates of metabolites, and also intracellular fluxes when the former are not enough.

The determination of metabolic flux distribution in undetermined systems may also be obtained by flux-balance analysis (FBA) [2]. In FBA, unknown fluxes are determined by linear programming (LP), whereby a given objective function, related to a given cellular physiological state, is optimised. Typically, the maximisation of the growth flux defined in terms of biosynthetic requirements is used as the objective function [2-5]. In general, FBA provides flux distribution for a desirable physiological state, it is however uncertain that the provided solution is unique [6]. Frequently multiple optima are obtained which are a consequence of the existence of redundant pathways in the metabolic network conferring structural robustness to cells [7].

Metabolic Pathway Analysis (MPA) is another flux-based analysis method. MPA, unlike FBA, do not look only at the properties of solutions selected by the statement of an objective, but study the full range of achievable biochemical network states that are provided by the solution space. Network-based MPA has focused on two approaches, elementary modes (EMs) and extreme pathways (EPs) [8-10]. These approaches are very similar being EPs a subset of EMs. In certain network topologies the sets of EMs and EPs coincide. They are both unique for a given network and can be considered as nondecomposable steady state flux distributions using a minimal set of reactions. The difference is that EP analysis decouples all internal reversible reactions into two separate irreversible reactions (forward and backward directions) and EMs analysis accounts for reaction directionality. In this work we have adopted the EMs concept since it has broad application; EPs analysis can exclude important routes of the network giving misleading results [10]. MFA focuses on single flux distributions, but in a complex metabolic network of reactions there is a space of admissible flux distributions. The MPA allows the transition from a reaction based perspective to a pathway-oriented view of metabolism because each feasible steady state flux distribution can be represented as a nonnegative combination of EPs or EMs [8,11].

Although flux-based analysis methods have been mainly used for metabolic engineering [1,12], they may also be useful in other phases of the bioprocess development cycle, namely for advanced bioreactor monitoring and control [13,14]. The EMs concept is particularly attractive since it reduces network complexity to a minimal set of reactions. Provost and Bastin [13] exemplified the use of the EMs concept for dynamic modeling of a CHO culture. The main objective of the present study is to derive an optimal control method that incorporates the knowledge of the metabolic network of the biological system under study using the EMs technique. Model-based off- and on-line control techniques are today well established in both theoretical and practical terms, and have been widely applied for bioprocess optimisation (e.g. [15,16]). The success of such methods is critically dependent on the quality of the supporting mathematical model. Not only accuracy in describing previously measured data but mainly the capacity to predict behaviour in unexplored states is the key for success. In previous studies [17,18], an iterative batch-to-batch optimisation scheme was developed and applied to the optimisation of recombinant BHK-21 cultures. The method is based on the premise that in general the biological system under consideration is only partially known or even poorly known in a mechanistic sense. Following this principle, a flexible hybrid parametric/nonparametric representation of the biological system was adopted to support the batch-to-batch optimisation scheme. It was verified that the model generalization capacity increases as more reliable mechanistic knowledge of cells is incorporated in the hybrid model. The algorithm presented here is essentially an extension to the previous batch-to-batch optimisation scheme whereby the knowledge concerning the metabolic network is incorporated in the optimisation algorithm. The methods will be exemplified through the application to a recombinant BHK-21 culture expressing the fusion glycoprotein IgG1-IL2.

## Results and discussion

### Proposed methodology

**Figure 1:**
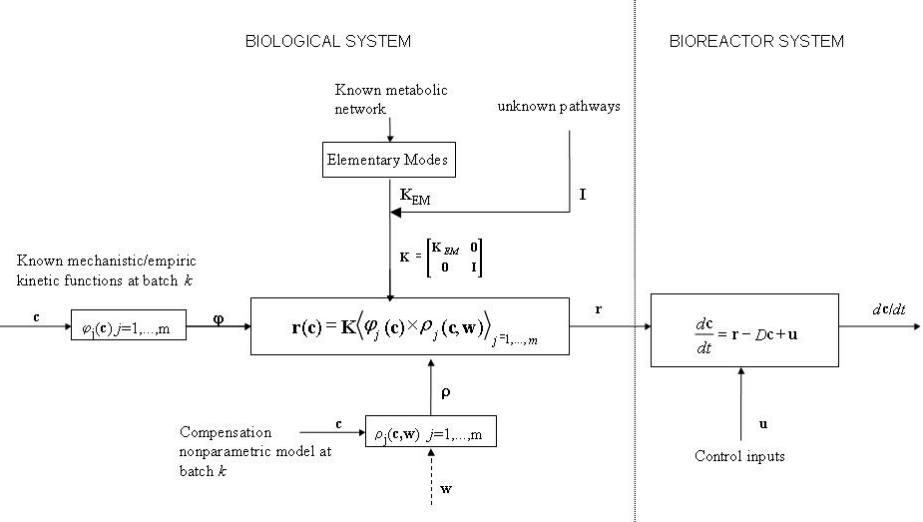
**General hybrid structure for bioprocesses**. This hybrid model structure integrates knowledge concerning the metabolism, transport phenomena and empirical process data. The bioreactor dynamics are then described by the material balance equations of each component occurring in the EMs. The EMs kinetics are identified with data from exploratory experiments, using chemometric techniques.

**Figure 2:**
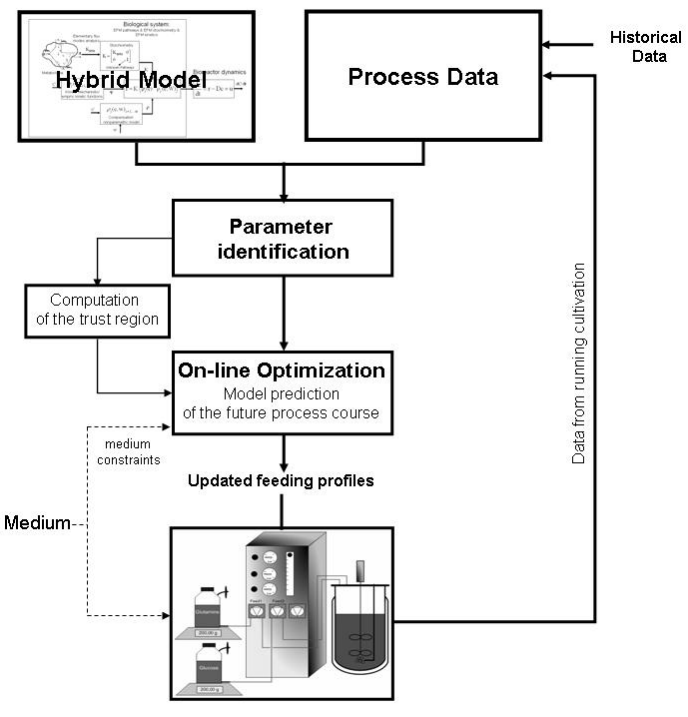
**Proposed model-based optimisation scheme**. On-line optimisation supported by the hybrid model. The process performance function includes a penalty term that accounts for the risk of model unreliability, i.e., extrapolation outside the model trust region. The parameter estimation as well as the optimisation of the future process course occurs every time a new measurement becomes available.

The proposed methodology for bioprocess monitoring and control is represented schematically in Figures 1, 2. The backbone of this methodology is the hybrid semiparametric model structure shown in Figure 1. The main design principle is flexible integration of knowledge concerning the metabolism, transport phenomena and empirical process data. The method contemplates the possibility of missing parts of the metabolism (e.g., the product metabolism) and of unknown reaction kinetics and underlying transduction mechanisms. Whenever knowledge is missing, empirical data modeling is called to fill the gaps. The starting point is the establishment of the metabolic network of the biological system under study. Firstly, the metabolic network is analyzed using the elementary modes technique. The overall network is decomposed into structural subunits, the EMs, which are the simplest paths connecting substrates with end-products. This structural analysis identifies all compounds (substrates, metabolites and products) taken up and/or secreted to the abiotic phase, which essentially define the system state space vector. The bioreactor dynamics are subsequently described by the material balance equations of each component occurring in the EMs. The EMs kinetics are identified with data from exploratory experiments, using chemometric techniques.

Once the model is properly validated, it can be used for on-line intracellular flux distribution monitoring and for on-line process performance optimisation (Figure 2). So the next step is the on-line implementation of the newly developed hybrid model for process monitoring and/or optimisation in the sense of maximizing the process performance by manipulating the control inputs, i.e., the optimal control problem [16,18-20]. The performance function includes a penalty term that accounts for the risk of model unreliability, i.e., extrapolation outside the model trust region. The empirical parameters are re-estimated, followed by the re-optimisation of the future process time course whenever new measurements of the process state are performed. In the lines below we describe in detail the steps involved.

#### Elementary modes analysis

**Figure 3:**
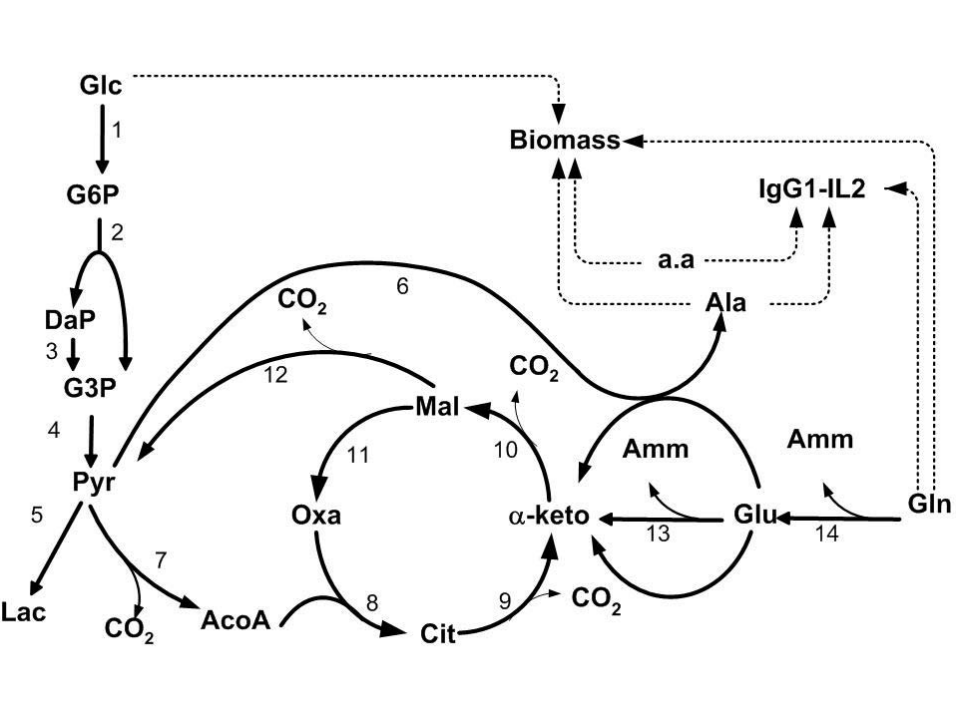
**BHK cells metabolic network**. The figure shows important pathways in the central metabolism of BHK cells. The dashed arrows indicate lumped pathways towards biomass and desired product synthesis.

We consider a generic metabolic network with *m *metabolites and *q *reactions such as the network represented in Figure 3. Assuming balanced growth and negligible dilution, the fundamental steady state mass balance equations on intracellular metabolites are expressed as follows:

{Nv=0vk>0     (1)
 MathType@MTEF@5@5@+=feaafiart1ev1aaatCvAUfKttLearuWrP9MDH5MBPbIqV92AaeXatLxBI9gBaebbnrfifHhDYfgasaacH8akY=wiFfYdH8Gipec8Eeeu0xXdbba9frFj0=OqFfea0dXdd9vqai=hGuQ8kuc9pgc9s8qqaq=dirpe0xb9q8qiLsFr0=vr0=vr0dc8meaabaqaciaacaGaaeqabaqabeGadaaakeaadaGabeqaauaabaqaceaaaeaaieqacqWFobGtcqWF2bGDcqGH9aqpcqWFWaamaeaacqWG2bGDdaWgaaWcbaGaem4AaSgabeaakiabg6da+iabicdaWaaaaiaawUhaaiaaxMaacaWLjaWaaeWaaeaacqaIXaqmaiaawIcacaGLPaaaaaa@3B17@

with **N **= {*n*_ij_} a *m *× *q *stoichiometric matrix and **v **= {*v*_j_} the vector of *q *metabolic fluxes with *v*_j _denoting the net specific rate of reaction *j*. Some of the *q *reactions are irreversible, thus the respective fluxes must be nonnegative: v_k _> 0 with *k *denoting the irreversible reactions in the metabolic network. The universe of solutions of system (1) forms a convex polyhedral cone in the solution space [21,22]. It is a property of this system that the infinite set of solutions forming the convex polyhedral cone may be expressed as nonnegative linear combinations of a finite set of *n *fundamental vectors e_i _called elementary modes (EMs):

v=∑i=1nλiei∀v∈ℜq     (2)
 MathType@MTEF@5@5@+=feaafiart1ev1aaatCvAUfKttLearuWrP9MDH5MBPbIqV92AaeXatLxBI9gBaebbnrfifHhDYfgasaacH8akY=wiFfYdH8Gipec8Eeeu0xXdbba9frFj0=OqFfea0dXdd9vqai=hGuQ8kuc9pgc9s8qqaq=dirpe0xb9q8qiLsFr0=vr0=vr0dc8meaabaqaciaacaGaaeqabaqabeGadaaakeaafaqabeqacaaabaGaemODayNaeyypa0ZaaabCaeaaiiGacqWF7oaBdaWgaaWcbaGaemyAaKgabeaakiabdwgaLnaaBaaaleaacqWGPbqAaeqaaaqaaiabdMgaPjabg2da9iabigdaXaqaaiabd6gaUbqdcqGHris5aaGcbaGaeyiaIiIaemODayNaeyicI4SaeyihHi8aaWbaaSqabeaacqWGXbqCaaaaaOGaaCzcaiaaxMaadaqadaqaaiabikdaYaGaayjkaiaawMcaaaaa@4706@

These elementary modes **e **= {e_i_} obey to constraints (1) and additionally to the elementarily constraint stating that there is no other non-null flux vector involving proper subsets of the reactions participating in that particular EM [22]. In the context of the present work, there are two most important features of the EMs analysis: it allows to identify all possible pathways for the conversion of substrates into products, and opens the way to the quantification of the relative importance of pathways at a given process stage. The non-null elements in each elementary mode, **e**_i_, define a subset of active reactions of the overall metabolic network **N**, which are essentially pathways for the conversion of substrates into products.

#### Hybrid dynamical model formulation

The knowledge acquired from the EMs analysis is integrated in the hybrid model structure represented in Figure 1. This structure allows the introduction of the *a priori *knowledge concerning the metabolic reactions and intracellular kinetics, but it is also open to the possibility of existing missing parts in both cases. The EMs analysis provides a stoichiometric matrix, **K_EM_**, that may be augmented (to a **K **matrix) if important parts of the metabolism are missing. For instance, the energetic and biosynthetic metabolism may be known, but product metabolism may be unknown. In this case the product concentration or other compounds and respective reactions kinetics are included through matrix **I **independently of the EMs.

Even though information about cellular components is growing rapidly, enzymes concentrations and intracellular kinetic data are difficult to obtain. In this work the EMs kinetics are defined by the product of two functions of the system state: a mechanistic/empirical function (in case it is available) and a nonparametric function that represents what is extracted from data without mechanistic interpretation. This model structure can be formulated mathematically by the following two equations, which may be regarded as a general hybrid model for ideal bioreactor systems [18]:

dcdt=r(c,w)−Dc+u     (3a)
 MathType@MTEF@5@5@+=feaafiart1ev1aaatCvAUfKttLearuWrP9MDH5MBPbIqV92AaeXatLxBI9gBaebbnrfifHhDYfgasaacH8akY=wiFfYdH8Gipec8Eeeu0xXdbba9frFj0=OqFfea0dXdd9vqai=hGuQ8kuc9pgc9s8qqaq=dirpe0xb9q8qiLsFr0=vr0=vr0dc8meaabaqaciaacaGaaeqabaqabeGadaaakeaadaWcaaqaaiabdsgaKHqabiab=ngaJbqaaiabdsgaKjabdsha0baacqGH9aqpcqWFYbGCcqGGOaakcqWFJbWycqGGSaalcqWF3bWDcqGGPaqkcqGHsislcqWGebarcqWFJbWycqGHRaWkcqWF1bqDcaWLjaGaaCzcamaabmaabaGaeG4mamJaeeyyaegacaGLOaGaayzkaaaaaa@4487@

**r**(**c**, **w**) = **K**⟨*ϕ*_*j*_(**c**) × *ρ*_*j*_(**c**, **w**)⟩_*j *= 1,...,*m *_   (3b)

with **c **a vector of *n *concentrations in the liquid phase, **r **a vector of *n *volumetric reaction rates, **K **a *n *× *m *coefficients matrix obtained from the elementary modes analysis, *ϕ*(**c**) are *m *kinetic functions established from mechanistic knowledge, *ρ*_*j*_(**c**,**w**) are *m *unknown kinetic functions, **w **a vector of parameters that must be estimated from data, *D *is the dilution rate, and **u **is a vector of *n *volumetric input rates (control inputs).

#### Identification of EMs kinetics

The reaction rate of elementary mode *j *is defined by the product *ϕ*_j_(**c**) × *ρ*_j_(**c**, **w**). The function *ϕ*_j_(**c**) represents "known" mechanisms whereas *ρ*_j_(**c**, **w**) represents unknown mechanisms. Redundancy and degeneracy are common problems in the determination of fluxes of biological networks [23,24]. It is very important to define *a priori *the conditions under which metabolic fluxes are identifiable. A rank of matrix K equal to the number of unknown EMs and an equivalent number of measured states are necessary conditions for the identifiability of system (3). If identifiable, the unknown functions *ρ*_j_(**c**, **w**) can be extracted from data using chemometric techniques such as multilinear regression, partial least squares, artificial neural networks and many other. In the frame of hybrid modeling, neural networks have been the most widely used technique for reaction kinetics modeling in biosystems [17,18,25-28]. We used a backpropagation neural network with a single hidden layer to define *ρ*_j_(**c**, **w**):

**ρ**(**c**, **w**) = **ρ**_max_*s*(**w**_2_*s*(**w**_1_**c **+ **b**_1_) + **b**_2_)     (4a)

s(x)=11+e−x     (4b)
 MathType@MTEF@5@5@+=feaafiart1ev1aaatCvAUfKttLearuWrP9MDH5MBPbIqV92AaeXatLxBI9gBaebbnrfifHhDYfgasaacH8akY=wiFfYdH8Gipec8Eeeu0xXdbba9frFj0=OqFfea0dXdd9vqai=hGuQ8kuc9pgc9s8qqaq=dirpe0xb9q8qiLsFr0=vr0=vr0dc8meaabaqaciaacaGaaeqabaqabeGadaaakeaacqqGZbWCcqGGOaakcqqG4baEcqGGPaqkcqGH9aqpdaWcaaqaaiabigdaXaqaaiabigdaXiabgUcaRiabbwgaLnaaCaaaleqabaGaeyOeI0IaeeiEaGhaaaaakiaaxMaacaWLjaWaaeWaaeaacqaI0aancqqGIbGyaiaawIcacaGLPaaaaaa@3E14@

with **ρ **= ⟨*ρ*_*j*_(**c**, **w**)⟩ a vector of *m *unknown reaction rates, **ρ**_max _a vector of scaling factors with dim(**ρ**_**max**_) = *m*, **w**_1_, **b**_1_, **w**_2 _and **b**_2 _are parameter matrices associated with connections between the nodes of the network, **w **is a vectored form of **w**_1_, **b**_1_, **w**_2_, **b**_2_, and s(·) the sigmoid activation function. A batch neural network training method was adopted, whereby the parameters **w **are estimated in the sense of least squares employing a quasi-Newton optimiser with gradients calculated by the sensitivities method [25,28,29] as described in the methods section.

#### Dynamic optimisation of culture operation: optimal control problem

In the dynamic optimisation step the process performance is optimised with respect to control inputs. This problem may be formulated mathematically as follows:

max⁡tb,c(0),u(t){J=f(c(tb))+∫0tbg(c(τ),u(τ))dτ}     (5)
 MathType@MTEF@5@5@+=feaafiart1ev1aaatCvAUfKttLearuWrP9MDH5MBPbIqV92AaeXatLxBI9gBaebbnrfifHhDYfgasaacH8akY=wiFfYdH8Gipec8Eeeu0xXdbba9frFj0=OqFfea0dXdd9vqai=hGuQ8kuc9pgc9s8qqaq=dirpe0xb9q8qiLsFr0=vr0=vr0dc8meaabaqaciaacaGaaeqabaqabeGadaaakeaadaWfqaqaaiGbc2gaTjabcggaHjabcIha4bWcbaGaeeiDaq3aaSbaaWqaaiabbkgaIbqabaWccqGGSaalieqacqWFJbWycqGGOaakcqaIWaamcqGGPaqkcqGGSaalcqWF1bqDcqGGOaakcqqG0baDcqGGPaqkaeqaaOWaaiWabeaacqqGkbGscqGH9aqpcqqGMbGzcqGGOaakcqqGJbWycqGGOaakcqqG0baDdaWgaaWcbaGaeeOyaigabeaakiabcMcaPiabcMcaPiabgUcaRmaapedabaGaee4zaCMaeiikaGIae83yamMaeiikaGIaeqiXdqNaeiykaKIaeiilaWIae8xDauNaeiikaGIaeqiXdqNaeiykaKIaeiykaKIaeeizaqMaeqiXdqhaleaacqaIWaamaeaacqqG0baDdaWgaaadbaGaeeOyaigabeaaa0Gaey4kIipaaOGaay5Eaiaaw2haaiaaxMaacaWLjaWaaeWaaeaacqaI1aqnaiaawIcacaGLPaaaaaa@66E5@

with *J *the performance index, t_b _the batch time, f(·) a terminal performance function and g(·) a time-dependent performance function. The algorithm used was the micro-genetic algorithm [30] coded by Carroll [31]. For simplicity, a piecewise constant approximation of the control inputs **u **was adopted. The optimisation (5) is subject to the constraint defined by the hybrid dynamical model (3)–(4) (and indirectly by the metabolic network (1)) but possibly also by other equality and inequality constraints regarding process states, cellular states and control inputs. Due to the use of nonparametric functions, namely of the neural network function (4), it is important to evaluate the unreliability risk during the optimisation. After the EM identification step, the measured input space is clustered by ellipsoidal functions (see the methods section). The clustered input space forms the trust region, wherein the function (4) is considered to be reliable. Optimisation (5) is then further constrained by the risk of function (4) inputs being outside the trust region. The technique is described in detail in the methods section.

### Case study: optimisation of recombinant BHK cultures

#### Process description

To illustrate the methodology described above it will be applied to a recombinant Baby Hamster Kidney (BHK-21A) culture expressing a fusion glycoprotein (an antibody type 1 linked to an interleukin type 2, IgG1-IL2) intended for cancer therapy [32]. The experiments were carried out in serum free and protein free medium (SMIF6, Life Technology, Glasgow, UK). The batch cultures were set up in a 2 1 reactor volume and the fed-batch cultures were set up at 3 different volume scales (2, 8 and 24 1). Sparger aeration was employed. Dissolved oxygen concentration was set at 15% of air saturation. Agitation rate used was 60 rpm; pH was set as 7.2 and controlled through the addition of CO_2_. Experimental data of viable cells concentration and six extracellular species (glucose, glutamine, lactate, ammonia, alanine and desired product) were collected. Analytical techniques are described elsewhere [17].

#### Elementary modes

**Figure 4:**
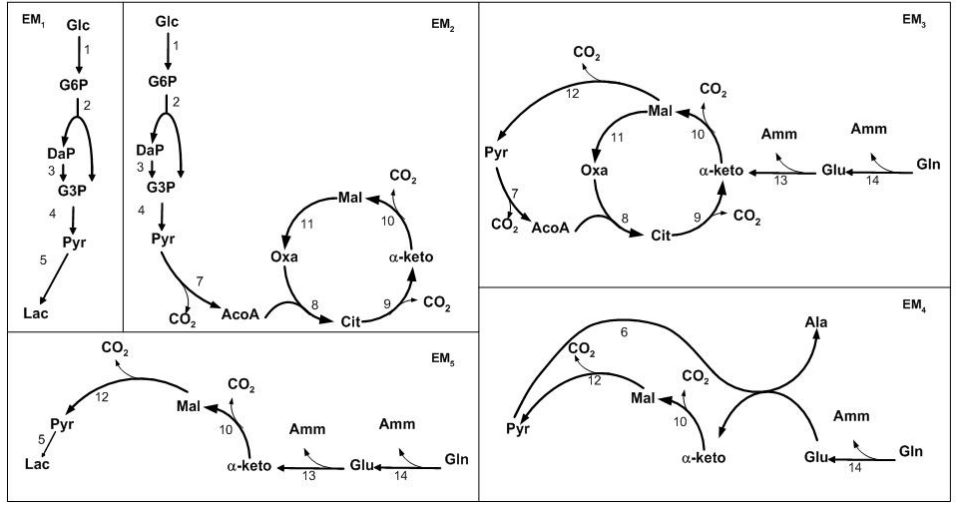
Elementary modes of the metabolic network considered.

BHK-21A cells use glucose and glutamine as major sources of carbon and energy, and produce lactate and ammonia as toxic by-products. A reduction of this waste production will improve both cellular growth and glycoprotein (IgG1-IL2) synthesis. Figure 3 shows the metabolic network adopted in this work [33,34]. As catabolic routes, the network includes the glucose and glutamine fluxes through glycolysis, glutaminolysis and TCA cycle. The amino acids metabolism was not considered; it was assumed that all of them are provided by the culture medium. The elementary modes of the 14 reactions that compose the catabolism were calculated using the *FluxAnalyser *program [22,35]. This system has five EMs, each one consisting of collections of reactions steps (Figure 4). The hypothesis of balanced growth allows the elimination of the intermediate metabolites resulting in a simplified set of reactions (see Table 1) connecting extracellular substrates (glucose and glutamine) with end-products (lactate, ammonia, alanine and carbon dioxide). The first elementary mode corresponds to the glucose flux converted into lactate; the second is the complete oxidation of glucose via TCA cycle (the most energetic pathway involving glucose); the third mode is the complete oxidation of glutamine (the most energetic pathway involving glutamine) and the fourth and fifth modes are partial oxidations of glutamine in alanine and lactate, respectively.

**Table 1:** Elementary modes of the metabolic network considered.

EM_1 _:	Glucose→ 2 Lactate
EM_2 _:	Glucose → 6 CO_2_
EM_3 _:	Glutamine → 5 CO_2 _+ 2 Ammonia
EM_4 _:	Glutamine → 2 CO_2 _+ Ammonia + Alanine
EM_5 _:	Glutamine → Lactate + 2 CO_2 _+ 2 Ammonia

#### Biomass and product synthesis

In addition to the catabolism, the anabolism and product synthesis must also be considered. For the sake of simplicity, the anabolism (biomass synthesis) was represented as a lumped equation combining the precursors of the main cellular building blocks (glucose, required for the synthesis of carbohydrates, lipids and nucleotides, and amino acids required for the synthesis of cellular proteins and some of them also for the synthesis of nucleotides). The stoichiometry established by Wei-Shou Hu and coworkers [36] for an hybridoma cell line was adopted in this work.

0.0208Glc + 0.0377Gln + 0.0133Ala + 0.0165Gly + 0.0096Val + 0.0133Leu + 0.0084Ile + 0.0033Met + 0.0081Pro + 0.0055Phe + 0.004Try + 0.0099Ser + 0.008Thr + 0.0Asn + 0.0077Tyr + 0.0004Cys + 0.0101Lys + 0.007Arg + 0.0033His + 0.026Asp + 0.0006Glu → Biomass    (7)

The IgG1-IL2 synthesis was also represented as a lumped equation as follows:

0.0104Gln + 0.0112Ala + 0.0139Gly + 0.0163Val + 0.0182Leu + 0.0061Ile + 0.0029Met + 0.0147Pro + 0.0072Phe + 0.0037Try + 0.0243Ser + 0.0163Thr + 0.0088Asn + 0.0077Tyr + 0.053Cys + 0.0136Lys + 0.0061Arg + 0.0043His + 0.0083Asp + 0.0096Glu → IgG1-IL2     (8)

This equation is based on the amino acid composition of the antibody IgG1 plus the interleukin IL2 (both amino acid sequences are available at [37]). The carbohydrate content of this fusion glycoprotein was neglected since the glucose contribution is extremely small compared to overall glucose consumption.

#### Hybrid model structure

The EMs analysis provides a simplified reaction mechanism based on which the following hybrid model structure (equivalent to system 3a-b) is formulated:

ddt[XvGlcGlnLacAmmAlaIgG]=[000010−1−100−0.0208000−1−1−0.0377−0.01042000000021000001−0.0133−0.0112000001][ρ1(Glc, Gln, Amm)⋅(XVGlc)ρ2(Glc, Gln, Amm)⋅(XVGlc)ρ3(Glc, Gln, Amm)⋅(XVGln)ρ4(Glc, Gln, Amm)⋅(XVGln)μ(Glc, Gln, Amm)⋅(XV)π(Glc, Gln, Amm)⋅(XVGln)]−D[XvGlcGlnLacAmmAlaIgG]+[0FGlcFGln0000]     (9)
 MathType@MTEF@5@5@+=feaafiart1ev1aaatCvAUfKttLearuWrP9MDH5MBPbIqV92AaeXatLxBI9gBaebbnrfifHhDYfgasaacH8akY=wiFfYdH8Gipec8Eeeu0xXdbba9frFj0=OqFfea0dXdd9vqai=hGuQ8kuc9pgc9s8qqaq=dirpe0xb9q8qiLsFr0=vr0=vr0dc8meaabaqaciaacaGaaeqabaqabeGadaaakeaadaWcaaqaaiabbsgaKbqaaiabbsgaKjabbsha0baadaWadaqaauaabeqaheaaaaqaaiabbIfaynaaBaaaleaacqqG2bGDaeqaaaGcbaGaee4raCKaeeiBaWMaee4yamgabaGaee4raCKaeeiBaWMaeeOBa4gabaGaeeitaWKaeeyyaeMaee4yamgabaGaeeyqaeKaeeyBa0MaeeyBa0gabaGaeeyqaeKaeeiBaWMaeeyyaegabaGaeeysaKKaee4zaCMaee4raCeaaaGaay5waiaaw2faaiabg2da9maadmaabaqbaeqabCGbaaaaaeaacqaIWaamaeaacqaIWaamaeaacqaIWaamaeaacqaIWaamaeaacqaIXaqmaeaacqaIWaamaeaacqGHsislcqaIXaqmaeaacqGHsislcqaIXaqmaeaacqaIWaamaeaacqaIWaamaeaacqGHsislcqaIWaamcqGGUaGlcqaIWaamcqaIYaGmcqaIWaamcqaI4aaoaeaacqaIWaamaeaacqaIWaamaeaacqaIWaamaeaacqGHsislcqaIXaqmaeaacqGHsislcqaIXaqmaeaacqGHsislcqaIWaamcqGGUaGlcqaIWaamcqaIZaWmcqaI3aWncqaI3aWnaeaacqGHsislcqaIWaamcqGGUaGlcqaIWaamcqaIXaqmcqaIWaamcqaI0aanaeaacqaIYaGmaeaacqaIWaamaeaacqaIWaamaeaacqaIWaamaeaacqaIWaamaeaacqaIWaamaeaacqaIWaamaeaacqaIWaamaeaacqaIYaGmaeaacqaIXaqmaeaacqaIWaamaeaacqaIWaamaeaacqaIWaamaeaacqaIWaamaeaacqaIWaamaeaacqaIXaqmaeaacqGHsislcqaIWaamcqGGUaGlcqaIWaamcqaIXaqmcqaIZaWmcqaIZaWmaeaacqGHsislcqaIWaamcqGGUaGlcqaIWaamcqaIXaqmcqaIXaqmcqaIYaGmaeaacqaIWaamaeaacqaIWaamaeaacqaIWaamaeaacqaIWaamaeaacqaIWaamaeaacqaIXaqmaaaacaGLBbGaayzxaaWaamWaaeaafaqabeGbbaaaaeaacqaHbpGCdaWgaaWcbaGaeGymaedabeaakiabcIcaOiabbEeahjabbYgaSjabbogaJjabbYcaSiabbccaGiabbEeahjabbYgaSjabb6gaUjabbYcaSiabbccaGiabbgeabjabb2gaTjabb2gaTjabcMcaPiabgwSixlabcIcaOiabbIfaynaaBaaaleaacqqGwbGvaeqaaOGaee4raCKaeeiBaWMaee4yamMaeiykaKcabaGaeqyWdi3aaSbaaSqaaiabikdaYaqabaGccqGGOaakcqqGhbWrcqqGSbaBcqqGJbWycqqGSaalcqqGGaaicqqGhbWrcqqGSbaBcqqGUbGBcqqGSaalcqqGGaaicqqGbbqqcqqGTbqBcqqGTbqBcqGGPaqkcqGHflY1cqGGOaakcqqGybawdaWgaaWcbaGaeeOvayfabeaakiabbEeahjabbYgaSjabbogaJjabcMcaPaqaaiabeg8aYnaaBaaaleaacqaIZaWmaeqaaOGaeiikaGIaee4raCKaeeiBaWMaee4yamMaeeilaWIaeeiiaaIaee4raCKaeeiBaWMaeeOBa4MaeeilaWIaeeiiaaIaeeyqaeKaeeyBa0MaeeyBa0MaeiykaKIaeyyXICTaeiikaGIaeeiwaG1aaSbaaSqaaiabbAfawbqabaGccqqGhbWrcqqGSbaBcqqGUbGBcqGGPaqkaeaacqaHbpGCdaWgaaWcbaGaeGinaqdabeaakiabcIcaOiabbEeahjabbYgaSjabbogaJjabbYcaSiabbccaGiabbEeahjabbYgaSjabb6gaUjabbYcaSiabbccaGiabbgeabjabb2gaTjabb2gaTjabcMcaPiabgwSixlabcIcaOiabbIfaynaaBaaaleaacqqGwbGvaeqaaOGaee4raCKaeeiBaWMaeeOBa4MaeiykaKcabaGaeqiVd0MaeiikaGIaee4raCKaeeiBaWMaee4yamMaeeilaWIaeeiiaaIaee4raCKaeeiBaWMaeeOBa4MaeeilaWIaeeiiaaIaeeyqaeKaeeyBa0MaeeyBa0MaeiykaKIaeyyXICTaeiikaGIaeeiwaG1aaSbaaSqaaiabbAfawbqabaGccqGGPaqkaeaacqaHapaCcqGGOaakcqqGhbWrcqqGSbaBcqqGJbWycqqGSaalcqqGGaaicqqGhbWrcqqGSbaBcqqGUbGBcqqGSaalcqqGGaaicqqGbbqqcqqGTbqBcqqGTbqBcqGGPaqkcqGHflY1cqGGOaakcqqGybawdaWgaaWcbaGaeeOvayfabeaakiabbEeahjabbYgaSjabb6gaUjabcMcaPaaaaiaawUfacaGLDbaacqGHsislcqqGebardaWadaqaauaabeqaheaaaaqaaiabbIfaynaaBaaaleaacqqG2bGDaeqaaaGcbaGaee4raCKaeeiBaWMaee4yamgabaGaee4raCKaeeiBaWMaeeOBa4gabaGaeeitaWKaeeyyaeMaee4yamgabaGaeeyqaeKaeeyBa0MaeeyBa0gabaGaeeyqaeKaeeiBaWMaeeyyaegabaGaeeysaKKaee4zaCMaee4raCeaaaGaay5waiaaw2faaiabgUcaRmaadmaabaqbaeqabCqaaaaabaGaeGimaadabaGaeeOray0aaSbaaSqaaiabbEeahjabbYgaSjabbogaJbqabaaakeaacqqGgbGrdaWgaaWcbaGaee4raCKaeeiBaWMaeeOBa4gabeaaaOqaaiabicdaWaqaaiabicdaWaqaaiabicdaWaqaaiabicdaWaaaaiaawUfacaGLDbaacaWLjaGaaCzcamaabmaabaGaeGyoaKdacaGLOaGaayzkaaaaaa@76DC@

The state space vector is formed by the concentrations of compounds that intervene in the final reactions set (glucose, Glc, glutamine, Gln, lactate, Lac, ammonia, Amm, alanine, Ala) and additionally the concentrations of viable cells, X_v_, and product, IgG:

**c **= [Xv, Glc, Gln, Lac, Amm, Ala, IgG]^T^.     (10)

Carbon dioxide was excluded because its concentration was not measured and because it doesn't interfere with the dynamics of the remaining variables (given that pH is controlled). The stoichiometric matrix, **K**, is established from the elementary modes of Table 1, but it also accounts for cell growth (5^th ^column) and product formation (6^th ^column) as lumped equations of glucose, glutamine and alanine. It should be noted that the 5^th ^EM was not included in K because preliminary results showed that this EM has negligible flux. This observation is in agreement with some published works [38-40], stating that lactate is mainly produced from glucose, being the percentage coming from glutamine very low (less than 10%). The volumetric reaction rates of the EMs were defined on the basis of the following assumptions:

(i) all reaction rates are specific (proportional to the concentration of viable cells),

(ii) the metabolic reactions considered are all irreversible (in this particular problem) and therefore the respective reaction rates are nonnegative

(iii) uncertainty in relation to kinetic constants and possible unknown saturation and inhibition effects.

(iv) only the concentrations of glucose, glutamine and ammonia have a significant impact on the specific reaction kinetics [41]. Lactate never reaches inhibitory levels in our experiments.

In the reaction rates of eq. (9) the term in parenthesis represents the *a priori *knowledge concerning the kinetics of the particular reaction (points (i) and (ii)) whereas the ρ_i_, μ and π terms represent the uncertainty concerning the reaction kinetics (point (iii)) and are functions of three state variables (point (iv)). With this particular formulation, the vector of known kinetic functions is given by:

ϕ(c) = [X_v_Glc X_v_Glc X_v_Gln X_v_Gln X_v _X_v_Gln]^T^,     (11)

whereas the vector of unknown kinetics is given by:

ρ = [ρ_1 _ρ_2 _ρ_3 _ρ_4 _μ π]^T ^= ρ(Glc,Gln,Amm,w)     (12)

The last term in eq. (9) is the control input vector **u **that in our case accounts for the volumetric feeding of glucose, *F*_*Glc*_, and glutamine, *F*_*Gln*_.

#### Identification of the EM kinetics

An important point for the identification of unknown flux functions (12) is that the rank of K is 6, thus the measurement of (10) (dim(**c**) = 7 > rank(K)) is sufficient for the observability of the EM kinetics. The other relevant point is the availability of sufficiently "rich" measurements to identify the "true" fluxes. Preliminary simulation tests showed that, for the system structure of eq (9), the "true" fluxes can be identified under typical fed-batch conditions (results not shown).

Experimental data of seven experiments (three batch and four fed-batch cultures) were used for the identification of the EM kinetics. Data of 5 experiments were used for parameter calibration whereas data of 2 experiments were used for model validation. The concentrations in the state space vector (eq. 10) were analyzed off-line according to methods published elsewhere [17]. The neural network has three inputs, [Glc,Gln,Amm]^T ^and 6 outputs as defined by eq. (12). The number of hidden nodes was tuned heuristically in the sense of minimizing the error of the validation data set. The best result was obtained with 5 hidden nodes giving a total number of network parameters equal to 56. The output scaling factors reflect the maximum kinetic rates and were defined as ρ_max _= [0.11 0.30 0.05 0.05 0.09 0.11]^T^.

**Figure 5:**
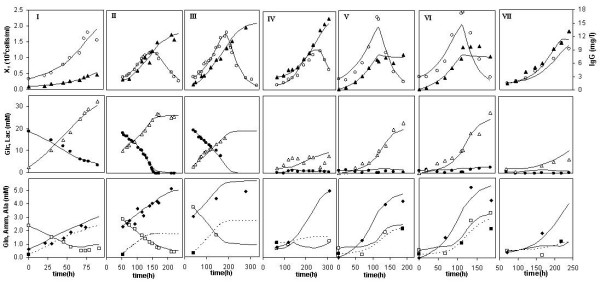
**Hybrid modeling results**. Modeling results of all seven state variables for both training (I-V) and validation data sets (VI-VII). Experiments I-III are Batch cultures and IV-VII are Fed-batch cultures.

**Figure 6:**
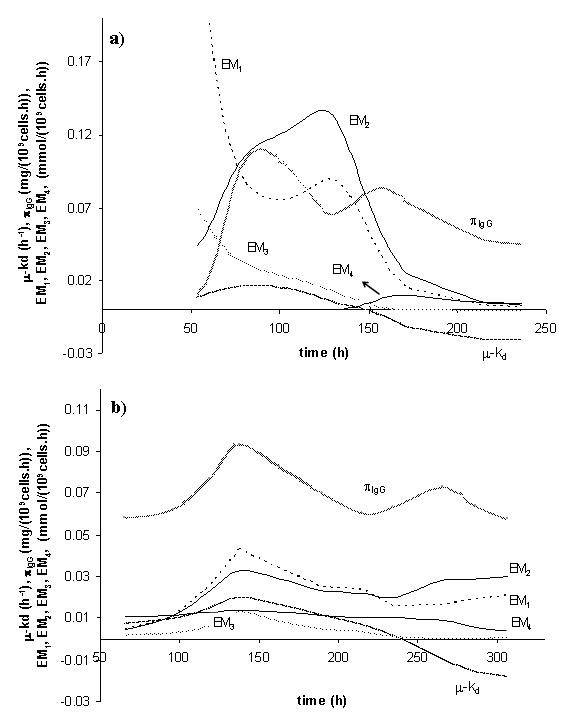
**Kinetic rates identified by the hybrid model**. The kinetic rates over the time course of bioreaction can provide valuable information concerning the evolution of BHK metabolism, (a) Batch culture; (b) Fed-batch culture.

The hybrid modeling results in terms of measured and predicted state variables are presented in Figure 5 for both training and validation data sets. Examples of EM kinetics identification are provided in Figure 6. The hybrid model was able to describe simultaneously all seven experiments with acceptable accuracy. In particular, the results with the validation data set strengthen the predictive potential of the model.

#### Metabolic interpretation

Figure 6 illustrates the EM kinetics identified by the hybrid model for two experiments, one batch and one fed-batch culture. The analysis of the flux distribution over the time course of bioreaction provides valuable information concerning the evolution of BHK metabolism and how to control the flux distribution through the feeding of glucose and glutamine.

In general, the metabolic activity of BHK cells during the cell growth phase is higher in the batch culture than in the fed-batch culture. The EM fluxes in the fed-batch experiments seem to be much more controlled than in the batch experiments. The high levels of glucose and glutamine at the beginning of the batch culture are mostly directed toward the overflow metabolism, i.e., waste production of lactate and alanine (EM_1 _and EM_4_). The fed-batch culture, which had lower glucose and glutamine concentrations during the cell growth phase, started with substantially lower consumption rates of these nutrients, while maintaining the flux to biomass.

Glucose is consumed for biomass synthesis and is metabolized through elementary modes EM_1 _and EM_2_. The consumption of this nutrient differs significantly from batch to fed-batch cultures. Cells use glucose in a more efficient way in fed-batch than in batch cultures particularly at the beginning of the culture, since the glucose metabolized to lactate (EM_1_) is much higher in the batch experiment. These results are in agreement with several published works for other mammalian cells [42-44], where it is reported that high glycolytic activity of animal cells results from high residual glucose.

Glutamine is consumed for product and biomass synthesis and is metabolized through elementary modes EM_3 _and EM_4_. The most energetic pathway involving glutamine, EM_3_, is practically inactive during the cell growth phase of the batch culture, being glutamine preferentially converted into alanine. On the opposite, this elementary flux mode is an important pathway in the course of the fed-batch culture, representing 50% of the total glutamine consumption. The higher production rate of alanine at high glutamine levels (such as the ones present at the beginning of batch cultures) is consistent with observations made by Doverskog et al. [44] and Vriezen et al. [45].

Concerning the specific formation rate of glycoprotein, π_IgG_, the product formation is consistently more stable in the fed-batch culture than in the batch culture. The product synthesis rate oscillates between 0,06 and 0,07 mg 10^-9 ^cells h^-1 ^in the former case whereas in the latter case the reaction is much lower in the beginning. This appears to be correlated with the overflow metabolism in the batch experiments, which seems to be detrimental for product synthesis.

#### On-line culture optimisation

The hybrid model was further used for on-line optimisation of a fed-batch BHK culture. The model parameters were re-tuned on-line using the data from the running cultivation. A batch training scheme was adopted, whereby the data of the running cultivation along with the data of historical experiments were used for model adjustment (Figure 2). Some variables, namely glucose, glutamine, lactate and viable cells concentration, can be measured off-line, with the results available after a short period of time (about 10 minutes). Therefore, at sampling times of 6 to 12 h, these measurements were stored in the training data set and then used for parameter identification using the same strategy previously described in the *Identification of the EM kinetics *section. The only difference was that the initial parameter values were those obtained in the off-line training procedure. The ammonia, alanine and IgG concentrations were exclude form this model adjustment step because they were quantified only at the end of the experiment.

After the parameters retuning step, the glucose and glutamine feeding rates were re-optimised (**u **= [F_GLC_, F_GLN_]^T^) in the sense of maximising the total amount of IgG1-IL2 produced at the end of the experiment (13).

max⁡uJ=CIgG−IL2(tf)V(tf)equation (9)0≤Fglu≤Fglu,max0≤Fgln≤Fgln,max(tf−t0)−1∫t0tfRISK[c(τ)]dτ≤RISKmax⁡     (13)
MathType@MTEF@5@5@+=feaafiart1ev1aaatCvAUfKttLearuWrP9MDH5MBPbIqV92AaeXatLxBI9gBaebbnrfifHhDYfgasaacH8akY=wiFfYdH8Gipec8Eeeu0xXdbba9frFj0=OqFfea0dXdd9vqai=hGuQ8kuc9pgc9s8qqaq=dirpe0xb9q8qiLsFr0=vr0=vr0dc8meaabaqaciaacaGaaeqabaqabeGadaaakeaafaqaaeqbcaaaaeaadaWfqaqaaiGbc2gaTjabcggaHjabcIha4bWcbaGaemyDauhabeaaaOqaaiabdQeakjabg2da9iabdoeadnaaBaaaleaacqWGjbqscqWGNbWzcqWGhbWrcqGHsislcqWGjbqscqWGmbatcqaIYaGmaeqaaOGaeiikaGIaemiDaq3aaSbaaSqaaiabdAgaMbqabaGccqGGPaqkcqWGwbGvcqGGOaakcqWG0baDdaWgaaWcbaGaemOzaygabeaakiabcMcaPaqaaaqaaiabbwgaLjabbghaXjabbwha1jabbggaHjabbsha0jabbMgaPjabb+gaVjabb6gaUjabbccaGmaabmaabaGaeGyoaKdacaGLOaGaayzkaaaabaaabaGaeGimaaJaeyizImQaemOray0aaSbaaSqaaiabdEgaNjabdYgaSjabdwha1bqabaGccqGHKjYOcqWGgbGrdaWgaaWcbaGaem4zaCMaemiBaWMaemyDauNaeiilaWccbiGae8xBa0Mae8xyaeMae8hEaGhabeaaaOqaaaqaaiabicdaWiabgsMiJkabdAeagnaaBaaaleaacqWGNbWzcqWFSbaBcqWFUbGBaeqaaOGaeyizImQaemOray0aaSbaaSqaaiabdEgaNjab=XgaSjab=5gaUjabcYcaSiab=1gaTjab=fgaHjab=Hha4bqabaaakeaaaeaacqGGOaakcqWG0baDdaWgaaWcbaGaemOzaygabeaakiabgkHiTiabdsha0naaBaaaleaacqaIWaamaeqaaOGaeiykaKYaaWbaaSqabeaacqGHsislcqaIXaqmaaGcdaWdXbqaaiabdkfasjabdMeajjabdofatjabdUealnaadmaabaacbeGae43yamMaeiikaGccciGae0hXdqNaeiykaKcacaGLBbGaayzxaaGaemizaqMae0hXdqNaeyizImQaemOuaiLaemysaKKaem4uamLaem4saS0aaSbaaSqaaiGbc2gaTjabcggaHjabcIha4bqabaaabaGaemiDaq3aaSbaaWqaaiabicdaWaqabaaaleaacqWG0baDdaWgaaadbaGaemOzaygabeaaa0Gaey4kIipaaaGccaWLjaGaaCzcamaabmaabaGaeGymaeJaeG4mamdacaGLOaGaayzkaaaaaa@AED9@

The optimisation (13) is subjected to the constraints of the process dynamics (equation 9), upper and lower bounds for the glucose and glutamine feeding rates, and the maximum risk of model unreliability, RISK_max _(see methods section). The increase in volume is negligible in this problem, thus it was not considered in optimisation (13). The risk constraint states that the average risk must not exceed a given maximum level defined by the user. This restricts the feasibility domain to low risk regions and is essential for process optimisation supported by hybrid models since the black-box model (4a,b) predictions degrade in regions of the input space with sparse measurements.

The micro-genetic algorithm coded by [31] was used to solve optimisation (13). The population size and number of generations was 5 and 2000, respectively. A maximum risk level of 35% (*RISK*_*max *_= 0.35) was adopted during the on-line optimisation experiment. At each sampling time, the flow rates of the feeding pumps were updated according to the re-optimised feeding profiles of glucose and glutamine.

**Figure 7:**
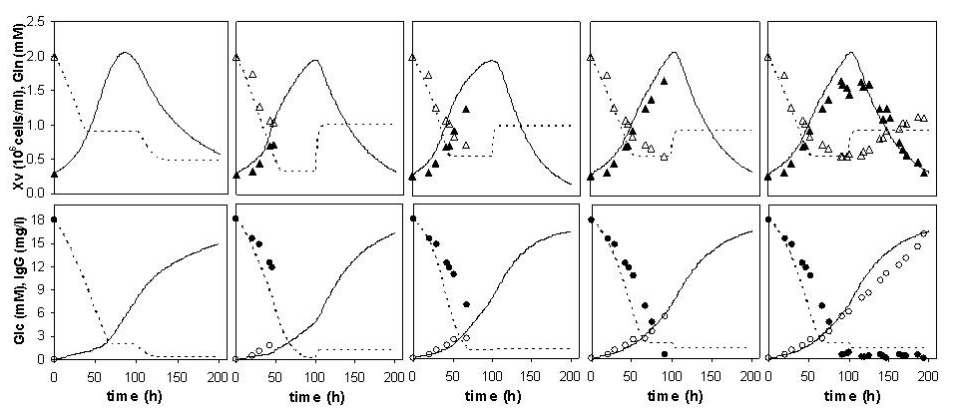
**Optimal trajectories during a fed-batch on-line optimization**. Optimised trajectories of process variables for five periods of cultivation: 0,46, 75, 92 and 195 h (lines are model predictions and symbols are experimental data).

Figure 7 shows the optimised trajectories and corresponding measurements for the main state variables (viable cells, glucose, glutamine and product) at cultivation times of 0 h (i.e., *a priori *optimised trajectories), 46 h, 75 h, 92 h and 195 h. The comparison of predicted and measured concentrations shows a very satisfactory performance for the on-line optimisation. Furthermore, although the measured product concentration was only available at the end of the experiment, the predicted time course of this variable follows closely the product measurements. The final product titre obtained was 16.4 mg/1 corresponding to a 10% increase in relation to previous experiments carried out with the same medium (initial glucose and glutamine concentrations) and without on-line optimisation.

#### Medium optimisation

**Figure 8:**
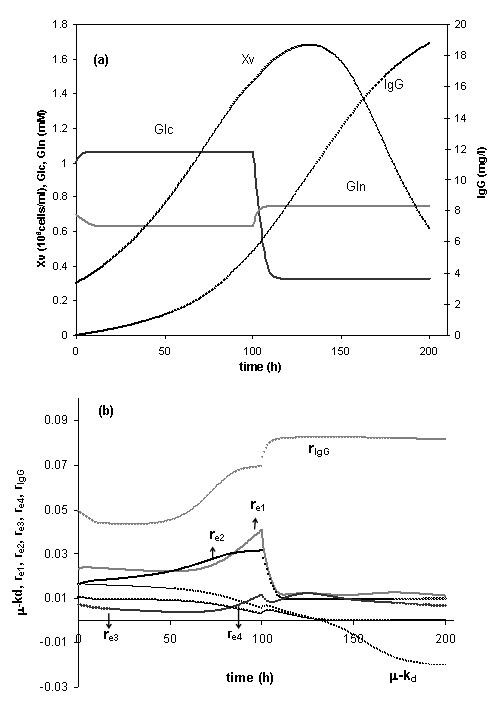
**Optimisation results with optimised medium**. (a) Predicted optimal trajectories of viable cells, glucose, glutamine and product concentrations starting with low levels of glucose and glutamine. (b) Distribution of intracellular elementary modes over the time course of the process for the optimal strategy.

Higher productivities are likely if the initial medium composition is optimised. The optimisation results of initial glucose and glutamine concentrations along with the corresponding feeding strategies are shown in Figure 8a. The medium should have initially low levels of glucose and glutamine. As for the feeding strategies, low levels of glucose and glutamine are optimal during the cell growth phase (glutamine concentration at 0.6 mM and glucose at 1 mM), whereas during the cell death phase, glutamine should slightly increase and glucose should decrease to a concentration of 0.4 mM. A significant increase of product titre was predicted under such cultivation conditions.

The kinetics of the elementary modes for the optimal strategy is presented in Figure 8b. The optimal strategy can be interpreted by means of intracellular flux distribution. The results were not much different from the elementary flux distribution of the fed-batch culture (Figure 6b). The main difference arises from the fact that the specific product formation (π _IgG_) increases during the cell death phase as a consequence of the increase of the glutamine concentration.

## Conclusion

The current work presents a hybrid modeling approach for bioprocesses that integrates information concerning intracellular metabolic fluxes, bioreactor transport phenomena and measured data. First, elementary flux analysis is used to reduce the biosystem metabolic network into a set of macroscopic reactions relating extracellular components only. A bioreactor dynamical model is then established from material balance equations of the compounds which intervene in the final reaction set. Nonparametric techniques are used for identification of the elementary modes kinetics from measured data. The method was successfully applied to a recombinant BHK-21A cell line producing a fusion glycoprotein. A significant result is the achievement of the flux distribution over the runtime of a bioprocess. The so obtained information can be used to identify conditions that favour product formation. A fed-batch BHK culture performed with on-line optimisation supported by the proposed methodology allowed a 10% increase in the final productivity. Higher productivities are expected if starting nutrients concentrations are optimised. The developed tool promises to be advantageous for optimising the productivity of fed-batch biochemical processes since the transfer and adaptation to different cell lines is reasonably straightforward

## Methods

### Neural network training procedure

A batch least squares criteria of residuals in concentrations was adopted

min⁡w{e=1P×n∑t=1P‖c¯(t)−c(t,w)‖2},     (M1)
 MathType@MTEF@5@5@+=feaafiart1ev1aaatCvAUfKttLearuWrP9MDH5MBPbIqV92AaeXatLxBI9gBaebbnrfifHhDYfgasaacH8akY=wiFfYdH8Gipec8Eeeu0xXdbba9frFj0=OqFfea0dXdd9vqai=hGuQ8kuc9pgc9s8qqaq=dirpe0xb9q8qiLsFr0=vr0=vr0dc8meaabaqaciaacaGaaeqabaqabeGadaaakeaadaWfqaqaaiGbc2gaTjabcMgaPjabc6gaUbWcbaacbeGae83DaChabeaakmaacmqabaGaeeyzauMaeyypa0ZaaSaaaeaacqaIXaqmaeaacqqGqbaucqGHxdaTcqqGUbGBaaWaaabCaeaadaqbdaqaaiqb=ngaJzaaraGaeiikaGIaeeiDaqNaeiykaKIaeyOeI0Iae83yamMaeiikaGIaeeiDaqNaeiilaWIae83DaCNaeiykaKcacaGLjWUaayPcSdWaaWbaaSqabeaacqaIYaGmaaaabaGaeeiDaqNaeyypa0JaeGymaedabaGaeeiuaafaniabggHiLdaakiaawUhacaGL9baacqGGSaalcaWLjaGaaCzcamaabmaabaGaeeyta0KaeGymaedacaGLOaGaayzkaaaaaa@59B9@

with *P *the number of measured patterns, *n *the number of state variables, c¯
 MathType@MTEF@5@5@+=feaafiart1ev1aaatCvAUfKttLearuWrP9MDH5MBPbIqV92AaeXatLxBI9gBaebbnrfifHhDYfgasaacH8akY=wiFfYdH8Gipec8Eeeu0xXdbba9frFj0=OqFfea0dXdd9vqai=hGuQ8kuc9pgc9s8qqaq=dirpe0xb9q8qiLsFr0=vr0=vr0dc8meaabaqaciaacaGaaeqabaqabeGadaaakeaaieqacuWFJbWygaqeaaaa@2E19@(*t*) and **c**(*t*,**w**) the measured and model state vectors. The concentrations were previously scaled by the measurements standard deviations. A quasi-Newton algorithm with conjugate gradient with line search (MATLAB™ optimisation toolbox) was adopted.

The gradients ∂e/∂**w **can be evaluated using the sensitivities equations, which can be obtained by differentiating eq. (3a) in order to **w**, yielding, after some manipulations, the following linear time-varying system:

ddt(∂c∂w)=A∂c∂w+B     (M2)
 MathType@MTEF@5@5@+=feaafiart1ev1aaatCvAUfKttLearuWrP9MDH5MBPbIqV92AaeXatLxBI9gBaebbnrfifHhDYfgasaacH8akY=wiFfYdH8Gipec8Eeeu0xXdbba9frFj0=OqFfea0dXdd9vqai=hGuQ8kuc9pgc9s8qqaq=dirpe0xb9q8qiLsFr0=vr0=vr0dc8meaabaqaciaacaGaaeqabaqabeGadaaakeaadaWcaaqaaiabbsgaKbqaaiabbsgaKjabbsha0baadaqadaqaamaalaaabaGaeyOaIylcbeGae83yamgabaGaeyOaIyRaee4DaChaaaGaayjkaiaawMcaaiabg2da9iab=feabnaalaaabaGaeyOaIyRae83yamgabaGaeyOaIyRaee4DaChaaiabgUcaRiab=jeacjaaxMaacaWLjaWaaeWaaeaacqqGnbqtcqaIYaGmaiaawIcacaGLPaaaaaa@466C@

with

A=K〈ρi∂ϕj∂c+ϕj∂ρj∂c〉−DIn,     (M3)
 MathType@MTEF@5@5@+=feaafiart1ev1aaatCvAUfKttLearuWrP9MDH5MBPbIqV92AaeXatLxBI9gBaebbnrfifHhDYfgasaacH8akY=wiFfYdH8Gipec8Eeeu0xXdbba9frFj0=OqFfea0dXdd9vqai=hGuQ8kuc9pgc9s8qqaq=dirpe0xb9q8qiLsFr0=vr0=vr0dc8meaabaqaciaacaGaaeqabaqabeGadaaakeaaieqacqWFbbqqcqGH9aqpcqWFlbWsdaaadeqaaGGaciab+f8aYnaaBaaaleaacqWGPbqAaeqaaOWaaSaaaeaacqGHciITcqGFvpGAdaWgaaWcbaGaemOAaOgabeaaaOqaaiabgkGi2kab=ngaJbaacqGHRaWkcqGFvpGAdaWgaaWcbaGaemOAaOgabeaakmaalaaabaGaeyOaIyRae4xWdi3aaSbaaSqaaiabdQgaQbqabaaakeaacqGHciITcqWFJbWyaaaacaGLPmIaayPkJaGaeyOeI0IaemiraqKae8xsaK0aaSbaaSqaaiabd6gaUbqabaGccqGGSaalcaWLjaGaaCzcamaabmaabaGaeeyta0KaeG4mamdacaGLOaGaayzkaaaaaa@52A7@

B=K〈ϕj∂ρj∂w〉,     (M4)
 MathType@MTEF@5@5@+=feaafiart1ev1aaatCvAUfKttLearuWrP9MDH5MBPbIqV92AaeXatLxBI9gBaebbnrfifHhDYfgasaacH8akY=wiFfYdH8Gipec8Eeeu0xXdbba9frFj0=OqFfea0dXdd9vqai=hGuQ8kuc9pgc9s8qqaq=dirpe0xb9q8qiLsFr0=vr0=vr0dc8meaabaqaciaacaGaaeqabaqabeGadaaakeaaieqacqWFcbGqcqGH9aqpcqWFlbWsdaaadeqaaGGaciab+v9aQnaaBaaaleaacqWGQbGAaeqaaOWaaSaaaeaacqGHciITcqGFbpGCdaWgaaWcbaGaemOAaOgabeaaaOqaaiabgkGi2kab=Dha3baaaiaawMYicaGLQmcacqGGSaalcaWLjaGaaCzcamaabmaabaGaeeyta0KaeGinaqdacaGLOaGaayzkaaaaaa@4276@

These equations must be integrated along with hybrid model eqs. (3)–(4). The initial value is (∂**c**/∂**w**)_t = 0 _= **0 **because the initial state is independent of parameters **w**. The evaluation of matrices **A **and **B **require the sensitivities ∂ϕ_j_/∂**c**, ∂ρ_j_/∂**w **and ∂ρ_j_/∂**c**. The first term is obtained by analytical derivation of known functions (in our case, by the derivation of eq. (9)). The other two matrices are obtained by backpropagating the identity matrix through the neural network. The backpropagation of a given identity matrix column 'i' results in the evaluation of vectors ∂ρi_j_/∂**w **and ∂ρi_j_/∂**c**.

### Evaluation of prediction risk

The trust region is the subspace of the input domain, where the model was properly validated with experimental data, showing low modeling error. Model predictions, c*, outside the trust region may have a high risk, RISK(c*), of being unreliable. The use of unreliable model predictions for process control should be avoided. For this reason, the value of the risk is used as a constraint to the optimisation (13). Here, the trust region was defined by *nc *ellipsoidal clusters of the form:

yC(c,mj,Σ)=e−0.5(c−mj)TΣ−1(c−mj)     (M5)
 MathType@MTEF@5@5@+=feaafiart1ev1aaatCvAUfKttLearuWrP9MDH5MBPbIqV92AaeXatLxBI9gBaebbnrfifHhDYfgasaacH8akY=wiFfYdH8Gipec8Eeeu0xXdbba9frFj0=OqFfea0dXdd9vqai=hGuQ8kuc9pgc9s8qqaq=dirpe0xb9q8qiLsFr0=vr0=vr0dc8meaabaqaciaacaGaaeqabaqabeGadaaakeaacqqG5bqEdaWgaaWcbaGaee4qameabeaakiabcIcaOGqabiab=ngaJjabcYcaSiab=1gaTnaaBaaaleaacqqGQbGAaeqaaOGaeiilaWccceGae43OdmLaeiykaKIaeyypa0Jaeeyzau2aaWbaaSqabeaacqGHsislcqaIWaamcqGGUaGlcqaI1aqncqGGOaakcqWFJbWycqGHsislcqWFTbqBdaWgaaadbaGaeeOAaOgabeaaliabcMcaPmaaCaaameqabaGaeeivaqfaaSGaeu4Odm1aaWbaaWqabeaacqGHsislcqaIXaqmaaWccqGGOaakcqWFJbWycqGHsislcqWFTbqBdaWgaaadbaGaeeOAaOgabeaaliabcMcaPaaakiaaxMaacaWLjaWaaeWaaeaacqqGnbqtcqaI1aqnaiaawIcacaGLPaaaaaa@568C@

with **m**_j _the cluster centres and **∑ **= diag{σ_i_^2^} the diagonal covariance matrix. The choice of the clusters number (*nc*) is done by trial and error according to the criteria of goodness of measurement density representation. The rule *nc *= *P*/3, with P the number of measured patterns normally provides acceptable results. The interpolation tolerances (**IT**) defines the distance to measured pattern such that *y*_*C *_> 0.5. The clusters width is then given by:

**σ **= [-2 ln(0.5/n)]^-1/2 ^**IT **    (M6)

The k-means algorithm was used to calculate **m**_j_. The final set of clusters forms a continuous density function f:**c**→v by applying the maximum operator:

v(c)=max⁡j{yC(c,mj,Σ)}.     (M7)
 MathType@MTEF@5@5@+=feaafiart1ev1aaatCvAUfKttLearuWrP9MDH5MBPbIqV92AaeXatLxBI9gBaebbnrfifHhDYfgasaacH8akY=wiFfYdH8Gipec8Eeeu0xXdbba9frFj0=OqFfea0dXdd9vqai=hGuQ8kuc9pgc9s8qqaq=dirpe0xb9q8qiLsFr0=vr0=vr0dc8meaabaqaciaacaGaaeqabaqabeGadaaakeaacqqG2bGDcqGGOaakieqacqWFJbWycqGGPaqkcqGH9aqpdaWfqaqaaiGbc2gaTjabcggaHjabcIha4bWcbaGaeeOAaOgabeaakmaacmqabaGaeeyEaK3aaSbaaSqaaiabboeadbqabaGccqGGOaakcqWFJbWycqGGSaalcqWFTbqBdaWgaaWcbaGaeeOAaOgabeaakiabcYcaSGGabiab+n6atjabcMcaPaGaay5Eaiaaw2haaiabc6caUiaaxMaacaWLjaWaaeWaaeaacqqGnbqtcqaI3aWnaiaawIcacaGLPaaaaaa@4BE1@

The output v(**c**) is a scalar between 0 and 1 that can be interpreted as the degree of membership of **c **to the data set used for training the black-box model (4). Low v values (i.e. **c **vectors out of the interpolation tolerance) are an indication of high risk of the black box model outputs being unreliable. Finally, the risk of black box model unreliability is given by: *RISK*(**c**) = 1 - *v*(**c**)     (M8)

## Abbreviations

BHK – Baby Hamster Kidney cells

CHO – Chinese Hamster Ovary cells

EM – Elementary Flux Mode

EP – Extreme Pathway

FBA – Flux Balance Analysis

IgG1-IL2 – Imunoglobulin type one linked to interleukin type two

LP – Linear Programming

MFA – Metabolic Flux Analysis

MPA – Metabolic Pathway Analysis

## Authors' contributions

All authors read and approved the final manuscript. RO developed the software. APT, CA and RO participated in the model implementation. APT performed experimental work. PA, MJTC and RO designed and coordinated the study. APT and RO drafted the manuscript.
